# Female Mate Choice Can Drive the Evolution of High Frequency Echolocation in Bats: A Case Study with *Rhinolophus mehelyi*

**DOI:** 10.1371/journal.pone.0103452

**Published:** 2014-07-30

**Authors:** Sébastien J. Puechmaille, Ivailo M. Borissov, Sándor Zsebok, Benjamin Allegrini, Mohammed Hizem, Sven Kuenzel, Maike Schuchmann, Emma C. Teeling, Björn M. Siemers

**Affiliations:** 1 Sensory Ecology Group, Max Planck Institute for Ornithology, Seewiesen, Germany; 2 School of Biology & Environmental Science, University College Dublin, Belfield, Dublin, Ireland; 3 Tabachka Bat Research Station, Tabachka, Bulgaria; 4 MTA-ELTE-MTM Ecology Research Group, Budapest, Hungary; 5 Naturalia environnement, Gallargues-le-Montueux, France; 6 Tunis Superior Institute for Biological Applied Sciences, Tunis, Tunisia; 7 Department Evolutionary Genetics, Max Planck Institute for Evolutionary Biology, Plön, Germany; Università degli Studi di Napoli Federico II, Italy

## Abstract

Animals employ an array of signals (i.e. visual, acoustic, olfactory) for communication. Natural selection favours signals, receptors, and signalling behaviour that optimise the received signal relative to background noise. When the signal is used for more than one function, antagonisms amongst the different signalling functions may constrain the optimisation of the signal for any one function. Sexual selection through mate choice can strongly modify the effects of natural selection on signalling systems ultimately causing maladaptive signals to evolve. Echolocating bats represent a fascinating group in which to study the evolution of signalling systems as unlike bird songs or frog calls, echolocation has a dual role in foraging and communication. The function of bat echolocation is to generate echoes that the calling bat uses for orientation and food detection with call characteristics being directly related to the exploitation of particular ecological niches. Therefore, it is commonly assumed that echolocation has been shaped by ecology *via* natural selection. Here we demonstrate for the first time using a novel combined behavioural, ecological and genetic approach that in a bat species, *Rhinolophus mehelyi*: (1) echolocation peak frequency is an honest signal of body size; (2) females preferentially select males with high frequency calls during the mating season; (3) high frequency males sire more off-spring, providing evidence that echolocation calls may play a role in female mate choice. Our data refute the sole role of ecology in the evolution of echolocation and highlight the antagonistic interplay between natural and sexual selection in shaping acoustic signals.

## Introduction

Acoustic components of the call of a wide range of animals (mammals, birds, amphibians, reptiles, and insects) including humans have been shown to contain reliable indicators of body size [1]–[4] or body strength [5], which are used by females in the context of mate choice [2], [3]. In mammals for example, red deer (*Cervus elaphus*) females have been shown to prefer low frequency roars, which signal large males [6], and in humans, women prefer lower pitched male voices, especially when they are close to the ovulation period [7]. When the signal is used for more than one function, antagonisms amongst the different signalling functions may constrain the optimisation of the signal for any one function, which can result in conflict amongst the functions and in the evolution of non-optimal signals [8], [9]. Echolocating bats represent a fascinating group in which to study the evolution of signalling systems as unlike bird songs or frog calls, echolocation has a dual role in foraging and communication [10], [11]. The function of bat echolocation is to generate echoes that the calling bat uses for orientation and food detection. Therefore, it is commonly assumed that echolocation has been shaped by ecology *via* natural selection alone [12], [13]. It is well known that call characteristics in the frequency domain (i.e. peak frequency, bandwidth) or time domain (i.e. pulse duration, interval between pulses) are directly related to the exploitation of particular ecological niches [12]–[14]. However, to date, no study has yet experimentally investigated the role of echolocation calls in the context of sexual selection in bats, despite their role being repeatedly suggested and discussed [15]–[18].

As bats are mostly active at low light levels where visual signals are difficult to perceive, other communication channels such as acoustic signals should play a large role in their mating systems [19]. Here we investigated whether cues contained in echolocation calls were used in a mate choice context by female *Rhinolophus mehelyi* (Mehely’s horseshoe bat), and found behavioural and genetic evidence that sexual selection may play a role in the evolution of echolocation calls. *Rhinolophus mehelyi* is an ideal species to investigate the role of sexual selection in the evolution of echolocation. Across the speciose Rhinolophidae family (n = 77) there is a negative correlation between echolocation peak frequency and size [20]. This allometric relationship is attributed to the physics of sound production, with larger bats having thicker vocal cords and/or larger resonance chambers and therefore, emitting lower frequencies [20]. However, *R. mehelyi* deviates from its congeners by having a peak frequency *ca.* 30 kHz higher than expected given its size (from the eqn. in Figure 1 in Ref. [20]). Here we investigated if this deviation from the echolocation/size norm may result from sexual selection.

**Figure 1:**
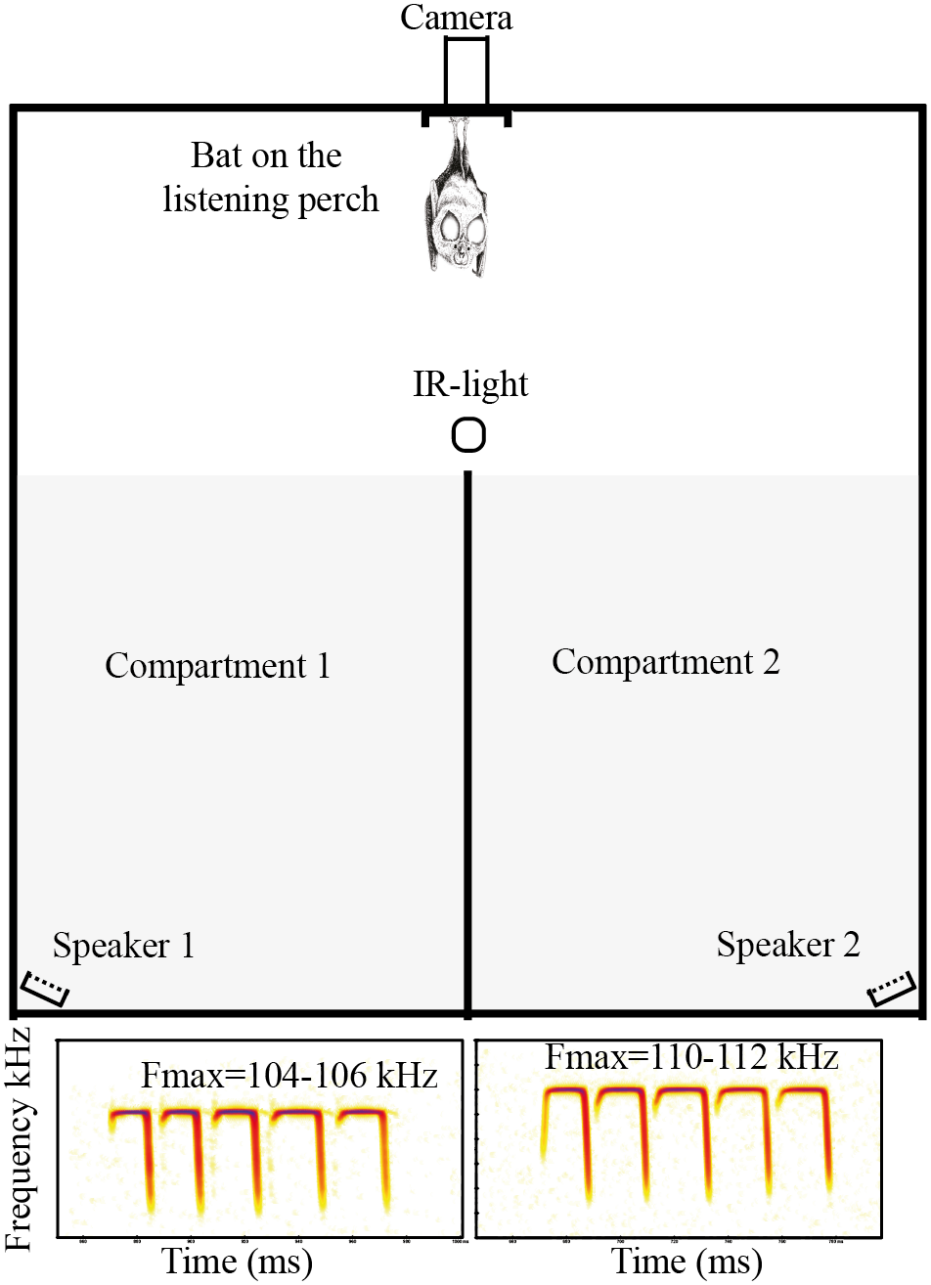
Experimental set-up for the mate choice experiment. Examples of spectrograms of low and high frequency echolocation calls played back are presented at the bottom of the figure.

We hypothesize that an individual’s frequency should be related to its body size and condition and hence represents an honest signal of mate quality. Females should therefore use the information conveyed in an individual’s frequency in a sexual selection context whereby females would choose their mates mainly on the basis of the frequency of their echolocation calls. Finally, we predict that males high peak frequency echolocation calls signal high quality mates, therefore males with higher peak frequency should have a higher reproductive success and therefore have on average a higher relatedness with other members of the colony (i.e. would have more relatives). We tested these hypotheses in wild populations of *R. mehelyi* by combining ecological, behavioural and genetic approaches.

## Materials and Methods

### Ethics statement

*Rhinolophus mehelyi* is a protected species in Bulgaria and capture, husbandry, behavioural studies and sample collection were carried out under license given by the responsible Bulgarian authorities (MOEW Sofia and RIOEW Ruse; permits No. 193/01.04.2009 and No. 205/29.05.2009). Officials from the Bulgarian Ministry of Environment and Water (MOEW) inspected our work in accordance with Section 8, Article 23, Paragraph 3 and 4 of the Bulgarian Biodiversity Law. According to Bulgarian laws no further ethical approval by a committee is required for any of the above mentioned procedures which were in accordance with the species-specific recommendations of the Canadian Council on Animal Care on bats [21]. All bats were successfully released in good health (at or above capture weight) at the capture site after experiments were completed.

### Capture and husbandry of bats for the behavioural experiments

Bats were captured with a harp trap or hand net at two caves in North-Eastern Bulgaria (Orlova Tschuka and Zorovitza caves, Ruse district) and kept for a maximum of 2 days at the Tabachka Bat Research Station (TBRS) of the Sensory Ecology Group (Max Planck Institute, Seewiesen). Bats were housed in screened tents with free access to water. Temperature (25°C) and humidity (75%) were kept close to natural conditions typically found in Zorovitza cave [22]. Bats were hand-fed mealworms after all experiments.

### Peak frequency and its relationship with body size/condition

To characterize peak echolocation frequency of adult *R. mehelyi*, we combined previously published data from Orlova Tschuka and Zorovitza caves [22], [23] and new data (this study) from the same caves (total: n = 162 individuals). As body temperature slightly influences the peak frequency of an animal [24], [25], all recordings used were made in similar conditions when animals were fully active, hence body temperature is not expected to have influenced the measurement of peak frequency in the present study. The peak frequencies of male (mean: 108.7, range: 104.7–111.3, n = 98; combined data set) and female (mean: 108.4, range: 104.0–110.8, n = 64) *R. mehelyi* were not significantly different (Mann Whitney U test, *P* = 0.23) and had a similar distribution (Kolmogorov-Smirnov test, *P* = 0.58); hence the data sets were pooled for the tests on normality and skewness. To investigate the relationship between peak frequency and body size/condition, only the new data set from this study was used (n = 90) as some measurements from previously published data [22], [23] were not available (e.g. not collected). From the present study, a total of 90 individuals (75 females and 15 males) captured during the mating season (2^nd^–24^th^ September 2010) were recorded in a screened tent while animals were hanging around 1 m from the detector. Recordings were made from stationary individuals to obtain a measure of peak frequency not altered by Doppler shift [23]. Echolocation calls were recorded with a D1000X ultrasound detector (Pettersson Elektronik AB, Uppsala, Sweden; ±5 dB between 30 and 120 kHz) with a sampling rate of 384 kHz. Given that peak frequency measured over many calls from the same individual showed limited variation compared to the range of frequency used in the species [23], we measured ten good signal-to-noise ratio calls to determine an individual’s peak frequency, which was then considered as the arithmetic mean of these ten calls. Following this procedure, the peak frequency of each individual was estimated with precision (mean standard error = 0.018 kHz). Measurements were done with the package Seewave [26] v.1.6.5 in R [27] v.3.0.2. The ‘fpeaks’ function was used to search for the peak frequency in the frequency spectrum over the entire call (FFT size: 2048; window type: Hanning) provided by the ‘spec’ function.

The following mass and size characteristics were measured by the same person (IB) for each individual; capture weight, forearm length, and head-body length (from the tip of the snout to the anus, ventrally). The ‘scaled mass index’ (SMI), the predicted body mass when the head-body length is standardised, was used as a measure of body condition [28] with males and females treated separately. Adult and sub-adult individuals were treated together as sub-adults *R. mehelyi* have been shown to reach their adult size in August in Bulgaria [29]. The SMI was calculated in R using custom written scripts and following equation 2 in Peig & Green [28]. Mass and size characteristics from 266 individuals (male: 169; females: 97) captured in Northern Bulgaria were used to precisely estimate the scaling component b_SMA_ necessary to calculate the SMI. As there was no significant correlation between the logarithm of body weight and the logarithm of forearm length (data not shown), the SMI was only calculated with captured weight as the body mass variable and head-body length as the linear body measurement. As some of the data deviated significantly from normality (Shapiro-Wilk test; *P*<0.05), we used the nonparametric Spearman’s *rho* statistic to test for relationships (unilateral test) between variables of interest and peak frequency for each gender separately.

### Mate choice test

Experiments were conducted in a one cubic meter box (Figure 1) that was sufficient for *R. mehelyi* to fly in given its high manoeuvrability [30]. The box was lined with sound-absorbing foam and the bat was placed on a listening perch (a wooden basket) at an equidistant position from two speakers (custom made Polaroid loudspeaker, University of Tübingen, Tübingen, Germany) and its activity monitored *via* an infrared-sensitive camera coupled with an infrared illumination (Figure 1). Females were presented with playback echolocation calls of conspecific adult males, with low (104–106 kHz) or high (110–112 kHz) frequency, obtained from the Schuchmann & Siemers [31] study. Recordings from four high frequency and four low frequency adult males were used as playbacks. For each male, three files of one second were created by cutting one second periods from a long recording, resulting in 12 files for low frequency and 12 files for high frequency males. Files for adult males with low or high frequency were standardised with on average the same number of calls (mean = 29.2 calls/file). In total, the data set comprised 702 different calls. A custom written Matlab routine (The MathWorks Inc.) drew from these two sets of files at random and played back one second files alternating between the two speakers until each speaker had played back 100 files. The speakers were connected to the computer running Matlab *via* a two output PCMCIA card (DAQ™-6062E, National Instruments). Playback files were normalized such that the loudest call had an amplitude of about 60 dB sound pressure level at the perch of the test bat. This amplitude mimics echolocation calls of a bat hanging or passing at a few meters’ distance [31]. The frequency response of the combined recording and playback system was flat (±3 dB). Files were played back at a sampling rate of 400 kHz. For technical reasons, there was a pause of 120 ms between each playback, resulting in the experiment lasting 224 seconds per individual. Each animal was only tested once to ensure their reactions were spontaneous. The assignment of low or high frequency playback to the left or right speaker was randomised prior to each individual’s testing. A total of 31 adult females and 30 sub-adult females were tested. A smaller number of males (9 adults and 6 sub-adults) was also included in the experiment to test whether the response was only seen in females and therefore, likely corresponded to mate choice. A few seconds after the bat was positioned at the listening perch, the calls were played back. The number of times each individual landed in each compartment of the box (high *versus* low frequency; Figure 1) was counted and considered as an indication of preference. When a bat landed more often in one compartment compared to the other one, it was considered as making a choice for the calls played back in the compartment with more landings (cf. the ‘Permutation test’ section below for statistical testing). On the contrary, when a bat did not land in any compartment or landed an equal number of times in each compartment, it was considered as having no reaction or not choosing between the high and low frequency calls respectively. This experimental set up was chosen to minimise the number of stimuli presented to each animal and ensure a spontaneous reaction. Besides the randomisation procedures described above, no other specific controls were needed for this test as the bats were presented with two stimuli that only differed by the frequency of the calls and hence any preference for either side of the box could be directly associated with the difference in the stimulus presented. If the bats showed no interest to the stimuli or showed similar interest to either of the two stimuli, the number of times animals landed in each compartment of the box (high *versus* low frequency) should be totally random, which was not the case (see main text for detailed results and statistics). Although it’s been shown that horseshoe bats have a rich communication repertoire (e.g. [32]), recent work suggests that typical echolocation calls are used by males before copulation [33], supporting the use of echolocation calls as stimuli presented to the tested animals. Previous work has demonstrated that *R. mehelyi* was able to recognize the gender of conspecifics from their calls only, providing evidence that cues on the gender of the emitter are encoded in echolocation signals [19], hence we considered that the animals tested recognized acoustically the gender of the call emitter.

Finally, after the experiment, we confirmed that the side of the box (right or left) playing high frequency calls did not have an effect on the outcome of the test (no choice, preference for low or high frequency), neither for males nor females (Fisher exact test, *p* = 0.76 and *p* = 0.51 respectively).

### Permutation test

For each animal, we counted the number of landings in the high and in the low frequency compartments. We tested the null hypothesis that the number of landings in the two compartments was equally likely. The test was carried out by comparing the difference in the average number of landings in the high and in the low frequency compartment for the observed data set *versus* the null distribution estimated from 99999 data sets where the number of landings in the high and low frequency compartment were randomly permuted and individuals were drawn with replacement. The test was implemented in R via a custom written script.

### Sample collection and DNA extraction

*Rhinolophus mehelyi* of both sexes were sampled from two colonies (Zorovitza [Ruse district], n = 111 and El Haouariya [Nabeul governorate], n = 57; [34]). Bats were captured either with a hand net, while roosting during the day, or with a harp-trap on emergence at dusk. Two wing biopsies were taken and stored in pure ethanol or dried with silica-gel until extraction. DNA was extracted as in Puechmaille *et al.* [17]. Recordings of echolocation calls from these animals were also obtained as specified in the section ‘Peak frequency and its relationship with body size/condition’.

### Microsatellites development and genotyping

Genomic DNA was isolated from tissue of *Rhinolophus mehelyi* (University College Dublin tissue collection, sample SP.C.46) and its sister species *R. euryale* (University College Dublin tissue collection, sample SP.C.55) using a salt extraction protocol. The genomic DNA was processed according to the Rapid Library Preparation Method Manual provided by Roche with slight modification. The concentration of genomic DNA was 1 µg/µl and 1 bar nitrogen for 1 min was used for nebulization. The Multiplex Identifier (MID) Adaptors for Rapid Libraries (Roche) were used as adaptors. The DNA fragments were quantified using an Agilent 2100 Bioanalyzer. Finally, the individual samples were combined into a single DNA library pool. The concentration of the library was again determined using an Agilent 2100 Bioanalyzer prior to emulsion PCR and sequencing as recommended by Roche. In total, we obtained 209,567 reads. After quality checks, 87,811 and 33,504 could be attributed to *R. euryale* and *R. mehelyi* respectively based on the MID-tag. Reads that contained perfect dinucleotide, trinucleotide, and tetranucleotide tandem repeats that were at least 12 bp in length were extracted using a Perl script as detailed in Castoe *et al.* [35]. Reads with microsatellites containing flanking regions with high quality priming sites (referred to as PAL, ‘potentially amplifiable loci’[35]) were submitted in batches to a locally installed Primer 3 program [36]. This primer pair design process was automated *via* a Perl script detailed in Castoe *et al.* [35] using the same primer design criteria, except the melting temperatures that were set between 58–62°C (optimal at 60°C) and the amplicon length that was set to different values in separate runs (105–125; 180–220; 280–320; 380–420; 480–520). Running the script with different length enabled selection of loci with amplicon lengths that would not overlap, facilitating multiplexing of a maximum of loci. Outputs from primer 3 were summarised with custom written R scripts and a total of 51 primer pairs were selected based on the number of repeats (favouring repeats numbers >10 whenever possible) and repeats type (26 tetranucleotides, 12 trinucleotides and 13 dinucleotides) (GenBank accession No. KC908111–KC911274). The 102 primers were screened in the program Autodimer v.1 [37] for non-specific interactions. To evaluate amplification success and select polymorphic loci among those 51 loci, two individuals from each species (*R. mehelyi* and *R. euryale*) were screened with unlabelled primers in a 10 µL simplex reaction as in Puechmaille *et al.* [38], except for the annealing temperature that was set to 56°C. PCR products were then run on a 2% agarose gel and scored. Based on amplification success and variability, a subset of 18 loci was selected and optimised to amplify in a single reaction. To facilitate accurate genotyping, a pigtail was added to 14 reverse primers following recommendation by Brownstein *et al.* [39]. Except for some minor modifications detailed in brackets, the multiplex PCR mix (primers sequence and concentration reported in Table S1), multiplex PCR reaction (annealing temperature at 60°C) and product sizing protocols (internal lane standard: LIZ-600; Applied Biosystems) followed Puechmaille *et al.* [40]. Hardy-Weinberg equilibrium (HWe) and linkage disequilibrium were tested using Genepop v3.4 [41] and null allele presence using MICRO-CHECKER [42]. Tests were carried out on the two populations separately and corrected for multiple comparisons using sequential Bonferroni correction. Significant departures from HWe were found for the locus RE007 in both populations and for locus RE017 and RM025 in Zorovitza and El Haouariya respectively. No linkage disequilibrium was detected between any loci pairs after Bonferroni correction. Null alleles were detected at locus RE007 in both populations, at locus RE013 in Zorovitza and locus RM025 in El Haouariya. As a result, loci RE007, RE013 and RE017 in Zorovitza and loci RE007, RE017 and RM025 in El Haouariya were not used for relatedness estimates.

### Relatedness estimates

Males with high reproductive success will sire more offspring and have more relatives in a colony, hence their average relatedness with other members of the colony will be high. The situation is different for females as irrespective of their frequency, each female can have a maximum of one offspring per year, hence we expect a much weaker correlation (if any) between peak frequency and average relatedness in females. To provide evidence that females indeed choose their mate according to peak frequency, we investigated the relationship between mean relatedness and peak frequency. Relatedness between individual pairs within colonies was first estimated in the program Coancestry [43] using the Wang moment estimator [44]. For groups showing a significant correlation between the relatedness estimate and peak frequency (males in El Haouariya), we verified that the significant relationship was not sensitive to the relatedness estimator used. We therefore estimated relatedness with a second moment estimator, the Lynch and Li estimator [45], [46], and two likelihood estimators, the dyadic [47] and the triadic [48] likelihood estimators and then ran the correlations with these separate estimates. Correlations coefficients were run in R using a spearman rank correlation test.

## Results

**Figure 2:**
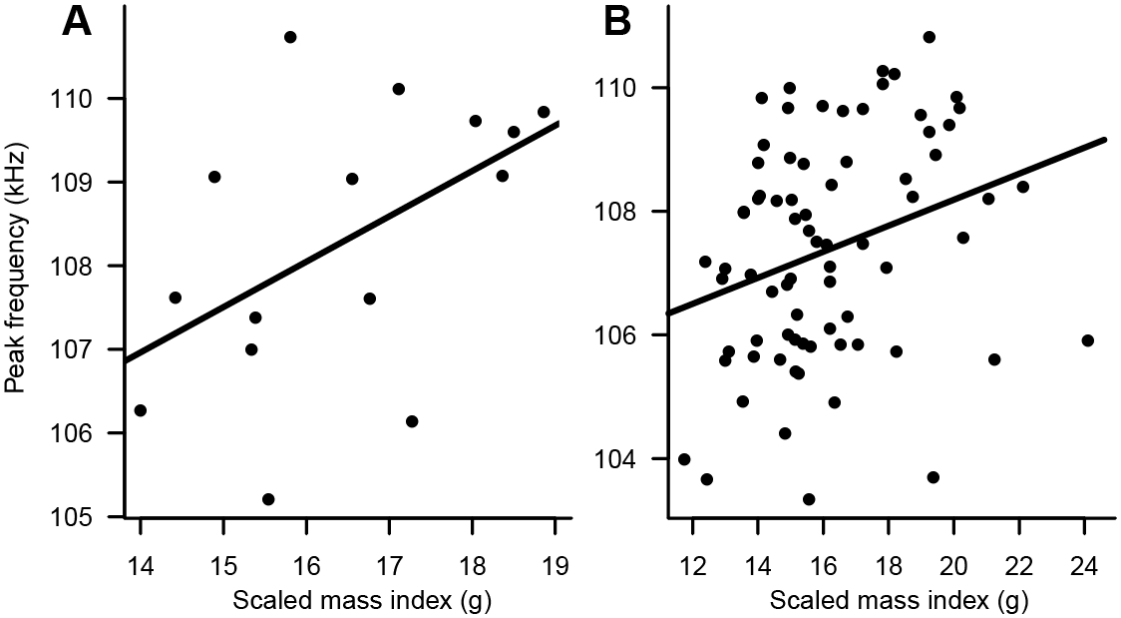
Echolocation peak frequency is an honest signal in *Rhinolophus mehelyi*. Correlation between peak frequency and body condition for (A), males and, (B), females captured during the mating season.

Based on wild caught animals, we demonstrated that body length and weight of individuals caught during the mating season showed a strong positive and significant correlation with peak frequency in males and females (Figure 2, Table 1; Spearman’s test, *rho* range: 0.48–0.73, *P*<0.05 for all relationships, *n* = 15 and *n* = 75 for males and females respectively) while forearm length did not (Table 1; Spearman’s test, *rho* = 0.04 and 0.16, *P*>0.05 in males and females). Body condition as measured by the scaled mass index standardized to the average head-body length also showed a similar positive and significant correlation with peak frequency in females and males (Figure 1; Spearman’s test, *rho* range 0.32–0.50, *P*<0.05 in both cases).

**Table 1:** Relationships between peak frequency and size, body weight and body condition for male and female *Rhinolophus mehelyi* captured in September, during the mating season.

	Males (n = 15)			Females (n = 75)		
	*Rho*	R^2^	*p*-value	*Rho*	R^2^	*p*-value
Freq. *vs.* forearm length (mm)	0.04	0.00	0.442	0.16	0.03	0.085
Freq. *vs.* body length (mm)	0.70	0.50	0.002	0.48	0.23	<0.001
Freq. *vs.* weight (g)	0.68	0.47	0.002	0.73	0.54	<0.001
Freq. *vs.* scaled mass index (g)	0.50	0.25	0.03	0.32	0.10	0.006

These positive relationships show that the peak frequency of an individual *R. mehelyi* is an honest signal for body size and body condition, larger and heavier individuals having higher frequencies.

**Figure 3:**
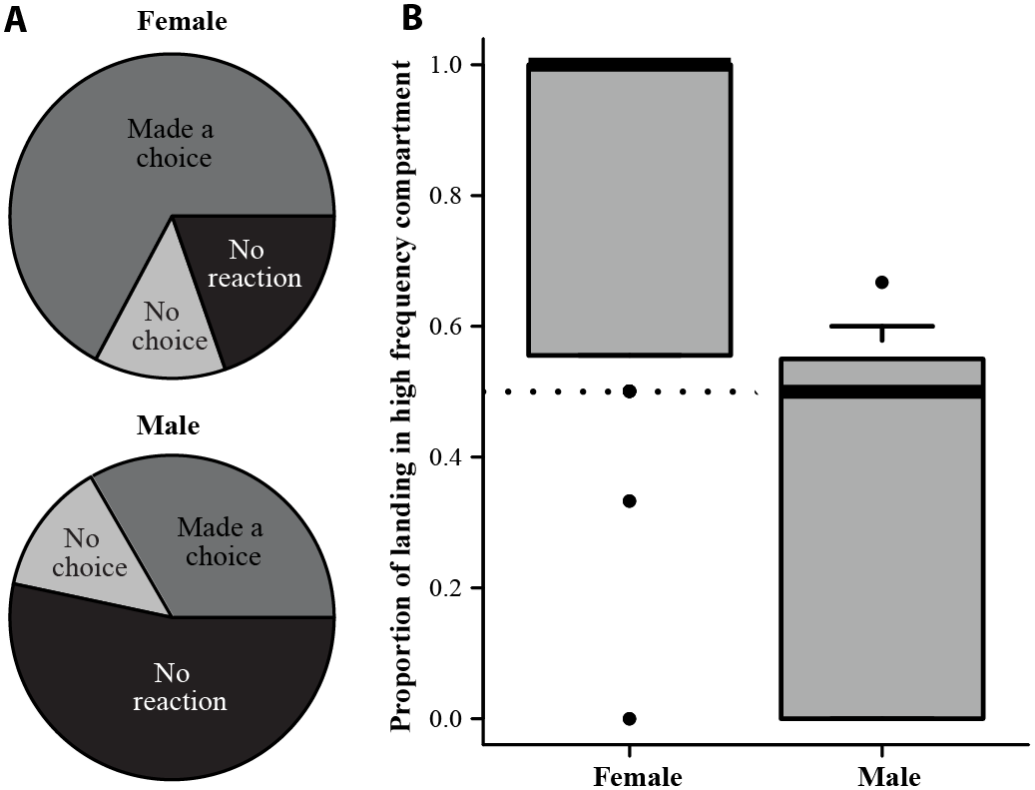
Behavioural experiments of mate choice preference in *Rhinolophus mehelyi.* (A) Depicts that females (top, n = 61) made a choice more often compared to males (bottom, n = 15) when confronted with adult male calls with high or low frequency. (B) Indicates that when a bat reacted to the test, females (left, n = 49) preferred high frequency calls while males (right, n = 7) did not show any preference (see text for statistical results). For each boxplot, the box represents the 25^th^ quantile, median (thick black bar) and 75^th^ quantile. To showcase possible outliers, whiskers were shortened to a length of 0.1 times the interquartile range.

In the behavioural experiments, females landed on average twice as often in the high frequency compartment compared to the low frequency compartment and this response was significantly different from random (permutation test; adults, n = 31, *P* = 0.002; sub-adults, *n* = 30, *P*<0.001). Age class of the tested animal did not significantly influence whether an individual would make a choice or not (Two-sided Fisher’s exact test, *P* = 0.42), nor the outcome of the choice (Two-sided Fisher’s exact test, *P* = 0.16), hence data from adult and sub-adult individuals were pooled for further tests. The results show that males were significantly less likely to make a choice compared to females (one-sided Fisher’s exact test, *n* = 15 and *n* = 61 for males and females respectively, *P* = 0.018; Figure 3A). When a choice was made, the sex of tested animals significantly affected the outcome of the choice (Fisher’s exact test, *P* = 0.03), demonstrating that males’ and females’ choices were different (Figure 3B). More specifically, the percentage of landings in the compartment where the high frequency adult male calls were played was significantly higher in females than in males (Mann Whitney U test, *P*<0.001; Figure 3B). Contrary to females, only a small proportion of males made a choice and these few individuals did not show any clear preference for high or low frequency males (three preferred low and two preferred high).

In *Rhinolophus* species, there is an acoustic fovea, which makes an individual’s hearing more sensitive to the frequency at which it echolocates. This could lead individuals to be passively attracted by the calls most like their own since they hear them best. Nevertheless, we found no relationship between female peak frequency and the proportion of females landing in the high frequency compartment (generalised linear model, binomial family, *P* = 0.79), suggesting that a female’s choice is active and not influenced by her hearing abilities (i.e. sensory bias).

**Figure 4:**
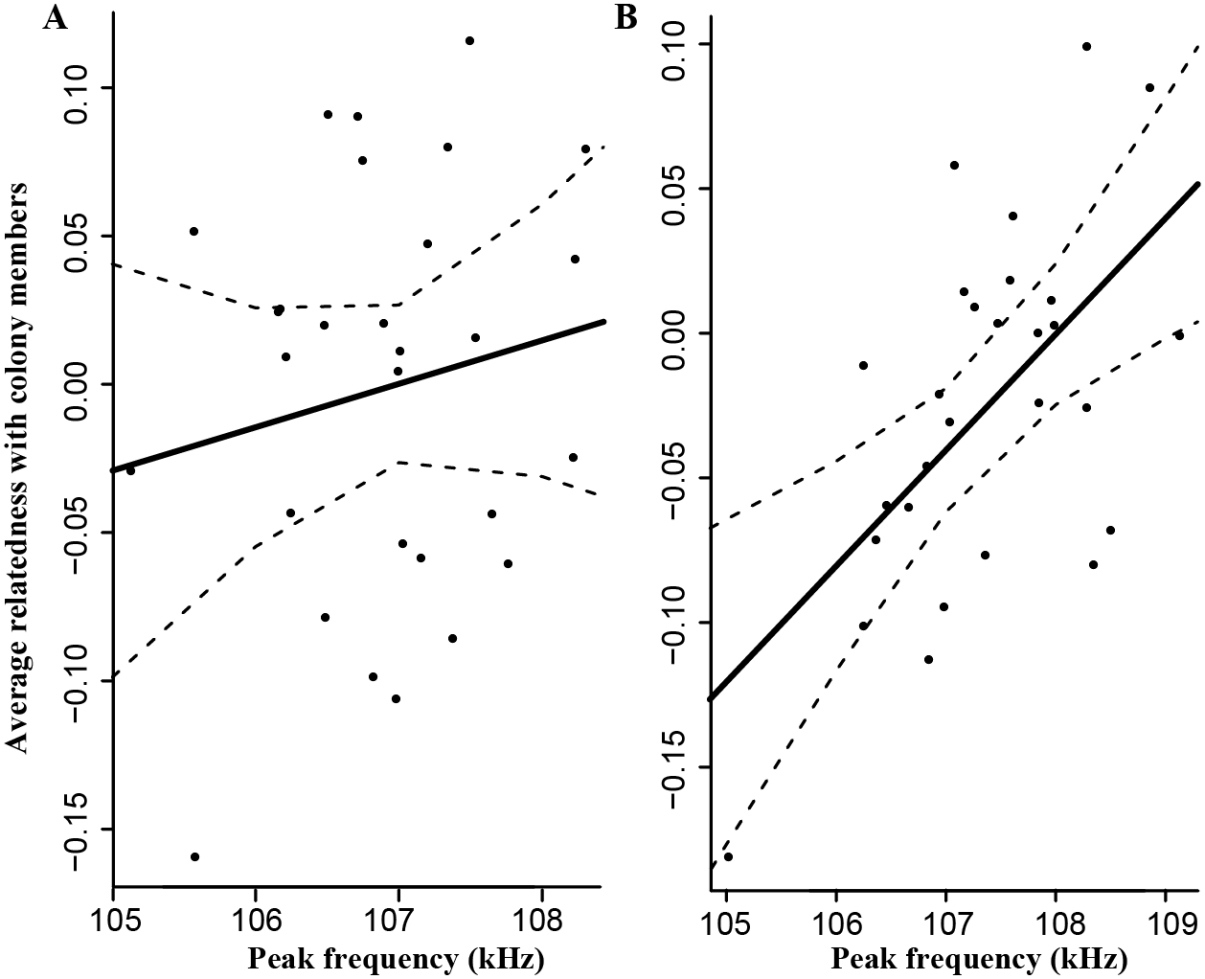
Correlation between an individual’s peak frequency and average relatedness with other members of the colony. Data are presented for (A), females (*n* = 29) and (B), males (*n* = 28) of El Haouariya colony (see text for statistical results). The solid back line represents a linear regression between peak frequency and relatedness and the dashed lines the 95% confidence interval of this regression.

To provide further data supporting that females indeed choose their mate according to peak frequency and specifically prefer males with high frequency, we investigated the relationship between a proxy of reproductive success (mean relatedness) and peak frequency. No significant relationship was found between the two variables in the *R. mehelyi* colony of Zorovitza (females, *n* = 57, *rho*  = 0.03, *P* = 0.48; males, *n* = 36, *rho*  = 0.05, *P* = 0.38) but this result was expected given the very low power of the test as only 2–3% of this large colony of *circa* 4000–5000 animals were sampled. To increase the power of the test, we therefore applied the same analysis to a smaller colony of *circa* 300 animals (El Haouariya colony) where *circa* 20% of the colony was sampled with similar number of males and females. As expected if echolocation frequency is playing a role in mate choice, we found no significant relationship between mean relatedness between colony members and peak frequency for adult females (*n* = 29, *rho* = 0.06, *P* = 0.38; Figure 4A) but we did find a strong and significant relationship for adult males (*n* = 28, *rho* = 0.47, *P* = 0.005; Figure 4B). This significant positive relationship in adult males was also obtained using three other relatedness estimates (Lynch & Li, Triadic and Dyadic likelihood; Spearman rank correlation test, *n* = 28, *rho* = 0.55, 0.41 & 0.31, *P* = 0.001, 0.015 & 0.05 respectively). In support of ongoing directional sexual selection for high call frequencies, we found that the distribution of echolocation frequencies was not normally distributed in adult individuals of the species (Shapiro-Wilk test, *P*<0.001) but was left-skewed (D’Agostino skewness test, *P* = 0.028).

## Discussion

By combining ecological, behavioural and genetic data, our study provides strong evidence that sexual selection plays a role in call frequency allocation, possibly against natural selection (see Text S1 & Figure S1). This novel finding is intriguing because it is commonly assumed that echolocation has been shaped by ecology *via* natural selection alone. Although research across many disciplines (e.g. [12], [49]) has greatly advanced our understanding of the echolocation system of bats it is still not proven that the high-frequency echolocation calls of *R. mehelyi* are truly deviating from their ecologically optimal peak frequency. Our current findings suggest that this could indeed be the case and will stimulate future studies to fully investigate this hypothesis.

Our data demonstrate that in *R. mehelyi*, unlike most other bat species investigated so far [50], peak frequency represent an honest signal for body size and body condition, larger and heavier individuals having higher frequencies. Similarly, strong correlations between male body mass or body condition and signal components have been reported in a wide range of animals [1]–[4]. Body condition at this period of the year (autumn) is very important for hibernating species (such as *R. mehelyi*) as an individual without enough fat reserves cannot make it through the winter, when there is no food available.

The theory of sexual selection predicts that females should preferentially mate with good quality males to generate more or fitter descendants [51], [52]. In this context, traits that reliably indicate good quality males or large males, such as echolocation call frequency, should be used by females to select mating partners. The results of the behavioural experiments obtained under controlled conditions support our prediction and demonstrate that during the mating season, female *R. mehelyi* are preferentially attracted to male calls with high frequency, which females might use as a proxy for male body size or body condition, as seen in a wide range of animals, including humans [2], [3], [6], [7], [53]. For example, studies on *Cervus elaphus* (red deer) have shown that hinds prefer roars indicating larger callers [6] potentially to gain indirect benefits of larger and more competitive offspring when mated. In our study, female preference for individuals with high peak frequency could be seen as a mechanism to avoid mating with sub-adults, which during the mating season (September) have a slightly lower average peak frequency than adults [23]. However, despite this slight difference, the overlap in peak frequency between adults and sub-adults is so large that a reliable determination of the age of an individual from its peak frequency is not possible ([23]; SJP, unpublished data). Therefore, avoidance of mating with sub-adults is an unlikely mechanism explaining female preference for individuals with high peak frequency.

Although on their own these experimental results are only suggestive of sexual selection acting on echolocation calls, they are further supported by field observations showing that during the mating season, males hang and display in caves and are visited by females that crawl from the main cluster [54], hence mate choice seems female based in a lek-like system. The heaviest *R. mehelyi* males pair most often with visiting females, whereas lighter males rarely do so [54]. This link between size and reproductive success has also recently been demonstrated in a closely related species, *R. ferrumequinum*, in which paternity success correlated positively with male forearm length [55].

The significant positive correlation between relatedness and peak frequency in males further supports the role of echolocation in mate choice, however these results could be interpreted as an ‘age effect’. If echolocation frequency increases with age, this correlation could have occurred as older males are more likely to have sired more offspring than younger males [55]. However, in male and female horseshoe bats, peak frequency only increases over the first year of life by ∼0.45 kHz; frequency then stays relatively stable over a lifetime with only a <0.09 kHz reduction in frequency on average per year [56]. Therefore, an ‘age-effect’ would result in a negative correlation between peak frequency and relatedness in animals older than one year [56], counter to what we observe. If age was primarily driving the correlation between relatedness and peak frequency, this correlation should also be observed in female bats, which was not the case. Therefore, this positive correlation cannot be due to an ‘age effect’. These results further support the conclusions of the behavioural experiments and provide evidence that on average, males with high frequency have a better reproductive success than other males.

Considered alone the ecological, behavioural and genetic studies are only suggestive of a role for echolocation in mate choice, however when considered together, these data provide the first documented indication that female bats choose their mate on the basis of echolocation frequency. The reproductive advantages the females gain from this selection process are not known but as male bats most likely only provide females with sperm [57], we hypothesise that the benefit of the female’s mate choice is indirect whereby their offspring will inherit “good genes” from the father and as a consequence, will have good body condition [58].

Traditionally it has been hypothesised that echolocation calls evolved under selection for optimal echo imaging, primarily for orientation and foraging and that call characteristics are heritable traits [12], [59], [60]. However, previous studies have revealed that the call frequency of *R. mehelyi* clearly deviates from the *Rhinolophus* genus trend by using a much higher frequency than expected for its size [23], resulting in a loss of prey detection distance [22]. *Rhinolophus mehelyi* is sympatric with another closely related but smaller species, *R. euryale*, which has an overlapping frequency range [23], [61]. Russo *et al.* [62] argued that in Sardinian *Rhinolophus* populations (including *R. euryale* and *R. mehelyi*), character displacement with respect to call frequency probably evolved to facilitate intraspecific communication and species recognition. It is therefore conceivable that ongoing sexual selection in *R. mehelyi* is driving character displacement. In support of ongoing directional sexual selection for high call frequencies, we showed that the distribution of echolocation frequencies was not normally distributed in the species but left-skewed. Although ecology surely remains a strong factor driving the evolution of echolocation calls *via* natural selection [12], our integrated multidisciplinary data highlight the role of communication and sexual selection in driving the evolution of echolocation calls.

Our study only tested sexual selection on echolocation calls as previously suggested [15]–[17], [63], [64] but given the rich repertoire of social calls in bats (e.g. [65]), it would also be very interesting to test the use of social calls in a sexual selection context [19]. Both, echolocation and social calls could be used concomitantly in a sexual selection context. Given that bats are able to precisely locate the source of acoustic signals, they are able to locate the emitter of the signal even in complete darkness, which is essential for mating. Once potential mates are close to each other, olfactory signals could play a role in the outcome of the encounter. The use of olfactory signals as honest signals in mate choice have been demonstrated in many animal taxa [66], though rarely in bats [67], which nevertheless have well developed olfactory receptor genes [68], [69]. It would be interesting to explore the use of olfactory signal in sexual selection in different bat species, including *Rhinolophus* species.

In summary, our results suggest that sexual selection plays a role in call frequency allocation, most likely against ecological selection pressures, hence both evolutionary forces must be jointly considered in the study of acoustic signalling [70]. These results highlight bats as a novel system in which to explore the interplay between natural and sexual selection on specific traits. The nature of this relationship has important consequences in understanding the evolution of animal communication systems, adaptation and speciation [71]. Given its dual role in foraging and sexual selection, echolocation can be considered as a ‘magic’ trait, which can be at the same time under divergent selection and causing non-random mating [72]. Echolocating bats therefore represent a group of choice to better understand speciation with gene flow [17] and the role of acoustic communication in evolution [70].

## Supporting Information

Figure S1 **Received relative echo levels from a target depending on distance to prey (x-axis) and frequency increase, when increasing echolocation frequency from an initial frequency of 80–110 kHz to 110 kHz.**
(PDF)

Table S1 **Characterisation of 18 microsatellites designed and tested in the present study.**
(PDF)

Text S1 **Why does** ***Rhinolophus mehelyi*** **echolocation call frequency deviate from the pattern predicted by allometry?**
(PDF)
