# Supplementary material for: Female Mate Choice Can Drive the Evolution of High Frequency Echolocation in Bats: A Case Study with Rhinolophus mehelyi
Source: PLoS One. 2014 Jul 30;9(7):e103452. doi: 10.1371/journal.pone.0103452 (PMC4116191; doi:10.1371/journal.pone.0103452)
Supplement: Figure S1 — Received relative echo levels from a target depending on distance to prey (x-axis) and frequency increase, when increasing echolocation frequency from an initial frequency of 80–110 kHz to 110 kHz. (PDF) [file pone.0103452.s001.pdf]

Supporting Figure S1

Female mate choice can drive the evolution of high frequency echolocation in bats: a case study with *Rhinolophus mehelyi*

Sébastien J. Puechmaille, Ivailo M. Borissov, Sándor Zsebok, Benjamin Allegrini, Mohammed Hizem, Sven Kuenzel, Maike Schuchmann, Emma C. Teeling and Björn M. Siemers

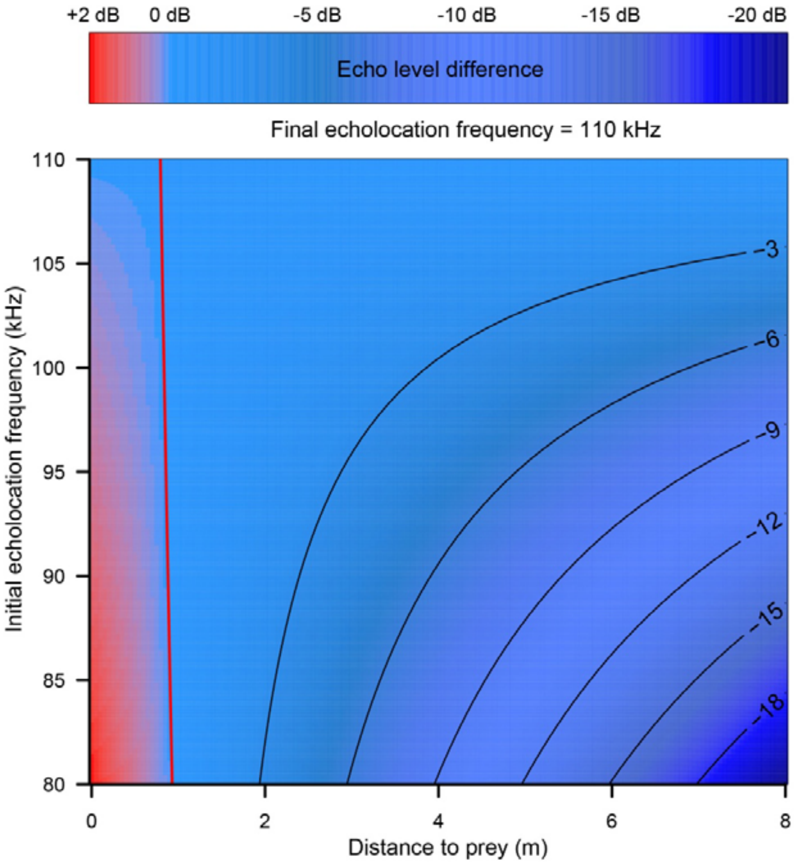

Figure S1. Received relative echo levels from a target depending on distance to prey (x-axis) and frequency increase, when increasing echolocation frequency from an initial frequency of 80-110 kHz to 110 kHz. Calculations take into consideration the opposite effects of increasing frequency: (a) an increase in directionality leading to an increase in source level and (b) an increase in atmospheric attenuation. The 0 dB line (in red) corresponds to the distance between the bat and its prey where the increase in source level is counteracted by an increase in atmospheric attenuation. The increase in frequency only results in a net gain of echo-level due to increase directionality at short distances (left of the 0 dB line). At longer distances (right of the 0 dB line), echoes are reduced because the effect of atmospheric attenuation outweighs the effect of increased directionality. For example, with an increase in frequency from 80 kHz to 110 kHz (x-axis), echoes returning from prey closer than 0.9 m from the bat are louder than if the bat had emitted calls at 80 kHz, but for prey further away than 0.9 m, the increase in atmospheric attenuation outweighs the increase in source level gained through greater directionality, hence the echoes are weaker. At 2.9 m, the echoes are twice as weak (- 6 dB). Calculations were made as in Jakobsen L., Ratcliffe J.M., Surlykke A. 2013 Convergent acoustic field of view in echolocating bats. *Nature* **493**, 93-96.
