# Supplementary material for: Female Mate Choice Can Drive the Evolution of High Frequency Echolocation in Bats: A Case Study with Rhinolophus mehelyi
Source: PLoS One. 2014 Jul 30;9(7):e103452. doi: 10.1371/journal.pone.0103452 (PMC4116191; doi:10.1371/journal.pone.0103452)
Supplement: Table S1 — Characterisation of 18 microsatellites designed and tested in the present study. (PDF) [file pone.0103452.s002.pdf]

Supporting Table S1

Female mate choice can drive the evolution of high frequency echolocation in bats: a case study with *Rhinolophus mehelyi*

Sébastien J. Puechmaille, Ivailo M. Borissov, Sándor Zsebok, Benjamin Allegrini, Mohammed Hizem, Sven Kuenzel, Maike Schuchmann, Emma C. Teeling and Björn M. Siemers

Table S1. Characterisation of 18 microsatellites designed and tested in the present study.

Locus	Repeat motif	Primer sequence (5'-3') (and 5' primer label or 'pigtail', which is underlined)	Primer (μM)	Genbank accession no.	Size range (bp)	Zorovitza (n=111)			El Haouariya (n=57)		
						n	H_O	H_E	*n*	H_O	H_E
RE001	(GGAA)18	F: NED-GTTTTCCCACCGAGAATGG	0.24	KC908929	284-324	10	0.82883	0.80547	8	0.75439	0.81757
		R: GTTTCTCTCTTAAATCAGGTGCC									
RE003	(AACC)16	F: NED-GAATTGATCAGCTTCACAGGC	0.08	KC908931	369-389	5	0.57658	0.49056	6	0.75439	0.72396
		R: GCTAGAGGAACTGCAATGTTAGC									
RE007	(TCTA)12	F: VIC-GTGTTCAGTGCTGCTATCCC	0.07	KC908936	117-145	8	0.42342	0.8499	6	0.5614	0.7533
		R: GTTTCTTATCGTGATCCATGCAAGC									
RE010	(AAAC)9	F: VIC-TTGGCTTGTTTGGAGAGTAGC	0.18	KC908955	479-495	5	0.51351	0.55615	2	0.40351	0.3827
		R: GTTTCTTGACAGCACTTCTTGCACAGC									
RE011	(AC)17	F: PET-TCCAAGAGTCACCATGAACC	0.15	KC908794	268-355	18	0.59459	0.59769	3	0.49123	0.49542
		R: ATTCCCAGCATCTGAACCC									
RE013	(ATT)14	F: VIC-GACAGGTGTAAGGTGCTATTTCG	0.27	KC908796	316-337	6	0.61261	0.6996	7	0.68421	0.71107
		R: GTTTCTTCCATCAACAAAGTGAAAAGGC									
RE015	(GGT)8	F: PET-GGGATTTGAGAGGTGTAGTGC	0.20	KC908814	488-491	2	0.01802	0.01794	1	0	0
		R: GTTTCTTGTGTTACTTCTCTCCTGGGG									
RE017	(GT)24	F: PET-CTGGACATCCTTTGCTTTCG	0.18	KC908111	183-216	9	0.68468	0.72761	10	0.87719	0.8744
		R: GTTTCTTCACACAGCAATGGAAACAGG									
RM002	(TTTC)18	F: VIC-AAAAGCTGTGCTCAAAGTGC	0.18	KC911030	364-372	2	0.06306	0.06135	3	0.47368	0.47493
		R: TCAAATCATGTTGCCTACAAGC									
RM003	(AAGG)16	F: PET-CAGAGTGGACCCTCAGTTCC	0.16	KC911031	392-448	11	0.93694	0.86959	12	0.89474	0.87254
		R: GTTTCTTACCTTGGCTCTAGAATGCCC									
RM006	(AAGG)15	F: FAM-CTGAGCTTCTGGAGGGGC	0.30	KC911034	484-526	13	0.81982	0.81546	11	0.85965	0.86524
		R: GTTTCCTTCTAACTATGGGTATATAAACTTGTGG									
RM010	(TAGA)13	F: NED-AATGTGTTGTCAGCCAGGG	0.50	KC911038	460-480	4	0.42342	0.41201	7	0.7193	0.72722
		R: GTTTCTTACCTAGTATGTTGCCATCTGTGG									
RM011	(TCTA)13	F: FAM-GATAGGCATGGAACATTGGG	0.24	KC911039	383-427	7	0.4955	0.48502	8	0.84211	0.77488
		R: TGTGCCTTAATATCTCATCGACC									
RM012	(TATC)13	F: FAM-GCAGCAAAGAGAAAAGACAGG	0.032	KC911040	178-180	2	0.51351	0.48253	2	0.15789	0.14672
		R: GTTTCTTCATCAAACAGTACCCGGC									
RM014	(TCCA)12	F: NED-GTGTCTGAATCCATCCTGGG	0.08	KC911042	176-188	3	0.47748	0.44633	4	0.33333	0.29312
		R: GTTTCTTGTGACACATAGCAAATGCTCC									
RM015	(ATGG)12	F: VIC-TTCAATCTGTTGAATGAGTGCC	0.07	KC911043	178-202	4	0.56757	0.53838	7	0.64912	0.69244
		R: CGACCCATCCACTTACTAATCC									
RM019	(GTG)15	F: FAM-TTTCAGAAATGCTATGTGAATGG	0.60	KC910743	262-271	2	0.01802	0.01794	2	0.2807	0.24344
		R: GTTTCACAAATGTTTATAGCAATTTTGTTTG									
RM025	(GT)21	F: PET-AGCCCTGGGTTCATCAGC	0.12	KC909042	110-140	11	0.7027	0.71909	8	0.40351	0.5274
		R: GTTTCTTAGTGTTAGTACACATCTCCCTCCC									

The number of alleles (*n*), observed (H_O) and expected (H_E) heterozygosities are reported for the two populations
