# Supplementary material for: Female Mate Choice Can Drive the Evolution of High Frequency Echolocation in Bats: A Case Study with Rhinolophus mehelyi
Source: PLoS One. 2014 Jul 30;9(7):e103452. doi: 10.1371/journal.pone.0103452 (PMC4116191; doi:10.1371/journal.pone.0103452)
Supplement: Text S1 — Why does Rhinolophus mehelyi echolocation call frequency deviate from the pattern predicted by allometry? (PDF) [file pone.0103452.s003.pdf]

Supporting Text S1

Female mate choice can drive the evolution of high frequency echolocation in bats: a case study with *Rhinolophus mehelyi*

Sébastien J. Puechmaille, Ivailo M. Borissov, Sándor Zsebok, Benjamin Allegrini, Mohammed Hizem, Sven Kuenzel, Maike Schuchmann, Emma C. Teeling and Björn M. Siemers

Supporting Text S1. Why does *Rhinolophus mehelyi* echolocation call frequency deviate from the pattern predicted by allometry?

R. mehelyi echolocates 30 kHz above what is expected given its size, which could allow the species to detect smaller prey size. Nevertheless, a frequency of 80 kHz (frequency expected for *R. mehelyi*) allows the detection of objects of 1.43 mm diameter [1], which is already much smaller than the typical prey size of *Rhinolophus* species, including *R. mehelyi* [e.g. 2]. For a frequency of 111 kHz (the maximum frequency observed in *R. mehelyi*), objects as small as 1.03 mm of diameter can be detected, however as they feed on larger prey this does not seem to have any advantageous effect in terms of prey size detection [1,3-6]. Additionally, higher frequencies attenuate more quickly in the air and increasing frequency from 80 kHz to 110 kHz results in a drastic decrease in detection distances. An increase in frequency leads to a more directional sound but the gain in intensity obtained *via* directionality is offset by attenuation for distance to objects of less than a meter (cf. Figure S1). This is in line with previous research which concluded that "*it becomes obvious that high-frequency calls of the bat* [*R. ferrumequinum*, frequency=83 kHz] *prove to be of minimal advantage in detecting small insects at short distances but of great disadvantage in detecting any insect at long distances. It follows that high-frequency calls cannot be considered as a specialization for small insects at short distances but as a restriction for any insect at long distance*" [1]. As *Rhinolophus* species of the size of *R. mehelyi* usually detect their prey from several meters away [7,8], having reduced intensity of echoes returning from those distances can be seen as a handicap as only prey that are very close to the bat will be detectable [9]. Hence the bats will have to fly for longer periods to acoustically scan the same space volume to find prey. This would be energetically very costly for *R. mehelyi* given that it faces a high flight cost as a consequence of its relatively high wing loading [10,11].The high

flight cost has been associated with *R. mehelyi*'s strategy of favouring perch-hunting rather than foraging on the wing, especially in the second half of the night [10,12,13].

High frequency calls could be seen as an advantage for clutter-feeders like a large majority of *Rhinolophus* species, whereby high frequency calls provide a more directional beam and hence would diminish echoes from objects situated to the side. Nevertheless, in the region studied (the Balkans) unlike most *Rhinolophus* species, *R. mehelyi* is not a clutter-feeder but more an open-space hunter foraging mainly in meadows, pastures and fields [12]. This tendency to forage in more open habitats has been associated with the co-occurrence with another competitor species, *R. euryale* [2,11,14], which is more abundant than *R. mehelyi* in the region studied [15]. It could however be argued that having a narrow beam shape at short distances could be an advantage by providing less background noise when approaching prey. Counter to this, recent research has demonstrated that *Rhinolophus* broaden their beam in the terminal phase of capture [8]. As the bats gets closer to the prey, the volume scanned by the echolocation beam reduces and the probability of the prey going off beam-focus increases, hence the bat uses a broader beam when situated less than one meter away from the prey [8].

Together, these ecological and acoustic findings provide arguments that from an ecological perspective, *R. mehelyi* peak frequency deviates from its local optima and would most likely benefit from having the lower peak frequency predicted by allometry. We argue that the high frequency echolocation calls observed in *R. mehelyi* are most likely driven by female mate choice rather than by ecology *via* natural selection alone.

References

1. Kober R, Schnitzler H-U (1990) Information in sonar echoes of fluttering insects available for echolocating bats. Journal of the Acoustical Society of America 87: 882-896. doi: 10.1121/1.398898

2. Salsamendi E, Garin I, Arostegui I, Goiti U, Aihartza J (2012) What mechanism of niche segregation allows the coexistence of sympatric sibling rhinolophid bats? Frontiers in Zoology 9: 30.

3. Sharifi M, Hemmati Z (2001) Food of Mehely's horseshoe bat *Rhinolophus mehelyi* in a maternity colony in western Iran. Myotis 39: 17-20.

4. Russo D, Jones G, Mucedda M (2001) Influence of age, sex and body size on echolocation calls of Mediterranean and Mehely's horseshoe bats, *Rhinolophus euryale* and *R. mehelyi* (Chiroptera: Rhinolophidae). Mammalia 65: 429-436.

5. Salsamendi E, Arostegui I, Aihartza J, Almenar D, Goiti U, et al. (2012) Foraging ecology in Mehely's horseshoe bats: influence of habitat structure and water availability. Acta Chiropterologica 14: 121-132. doi: 10.3161/150811012x654330

6. Salsamendi E, Garin I, Almenar D, Goiti U, Napal M, et al. (2008) Diet and prey selection in Mehelyi's horseshoe bat *Rhinolophus mehelyi* (Chiroptera, Rhinolophidae) in the south-western Iberian Peninsula. Acta Chiropterologica 10: 279-286.

7. Schuchmann M, Siemers BM (2010) Variability in echolocation call intensity in a community of horseshoe bats: a role for resource partitioning or communication? PLoS ONE 5: e12842. doi: 10.1371/journal.pone.0012842

8. Matsuta N, Hiryu S, Fujioka E, Yamada Y, Riquimaroux H, et al. (2013) Adaptive beam-width control of echolocation sounds by CF-FM bats, *Rhinolophus ferrumequinum nippon*, during prey-capture flight. The Journal of Experimental Biology 216: 1210-1218. doi: 10.1242/jeb.081398

9. Surlykke A, Kalko EK (2008) Echolocating bats cry out loud to detect their prey. PLoS ONE 3: e2036. doi: 10.1371/journal.pone.0002036

10. Voigt C, Schuller BM, Greif S, Siemers B (2010) Perch-hunting in insectivorous *Rhinolophus* bats is related to the high energy costs of manoeuvring in flight. Journal of Comparative Physiology B 180: 1079-1088. doi: 10.1007/s00360-010-0466-x

11. Salsamendi E, Aihartza JR, Goiti U, Almenar D, Garin I (2005) Echolocation calls and morphology in the Mehely's (*Rhinolophus mehelyi*) and mediterranean (*R. euryale*) horseshoe bats: implications for ressource partitioning. Hystrix, Italian Journal of Mammalogy 16: 149-158.

12. Dietz C (2007) Aspects of ecomorphology in the five European horseshoe bats (Chiroptera: Rhinolophidae) in the area of sympatry [PhD Thesis]. Tübingen: University of Tübingen. 237 p.

13. Dietz C, Von Helversen O, Nill D (2009) Bats of Britain, Europe & Northwest Africa. London: A & C Black Publishers Ltd. 400 p.

14. Russo D, almenar D, Aihartza J, Goiti U, Salsamendi E, et al. (2005) Habitat selection in sympatric *Rhinolophus mehelyi* and *R. euryale* (Mammalia: Chiroptera). Journal of Zoology 266: 327-332. doi: 10.1017/S0952836905006990

15. Benda P, Ivanova T, Horáček I, Hanák V, Červený J, et al. (2003) Bats (Mammalia: Chiroptera) of the Eastern Mediterranean. Part 3. Review of bat distribution in Bulgaria. Acta Societatis Zoologicae Bohemoslovenicae 67: 245-357.
